# Positive effects of selenized-oligochitosan on zearalenone-induced intestinal dysfunction in piglets

**DOI:** 10.3389/fvets.2023.1184969

**Published:** 2023-05-16

**Authors:** Shunyi Qin, Yukai Peng, Fuze She, Jianbin Zhang, Liuan Li, Fu Chen

**Affiliations:** ^1^Tianjin Key Laboratory of Agricultural Animal Breeding and Healthy Husbandry, College of Animal Science and Veterinary Medicine, Tianjin Agricultural University, Tianjin, China; ^2^Department of Agricultural Science and Technology, Hotan Vocational and Technical College, Hotan, People's Republic of China; ^3^College of Veterinary Medicine, Qingdao Agricultural University, Qingdao, China

**Keywords:** selenized-oligochitosan, zearalenone, piglets, digestion function, barrier function

## Abstract

This paper assessed the positive effects of selenized-oligochitosan (SOC) on zearalenone(ZEN)-induced intestinal dysfunction in piglets. Sixty piglets were randomly divided into 4 groups. Group C was fed the basal diet as a control and Group Z was supplemented with 2 μg/g ZEN in the basal diet; Group ZS1 and ZS2 were supplemented with 0.3 or 0.5 μg/g SOC (calculated by selenium), in addition to 2 μg/g ZEN in the basal diet. After 42 days, ileal mucosal structure, digestive enzyme activities, tight junction protein mRNA expressions, plasma D-lactate and D-xylose contents, and plasma diamine oxidase activities were determined. Compare with Group C, ileal villus height, value of villus height/crypt depth, trypsin, lipase and α-amylase activities, occluding, claudin-1 and ZO-1 mRNA expressions, and plasma D-xylose levels were significantly decreased (*p* < 0.01) in piglets of group Z; while compare to Group C, ileal crypt depth, plasma D-lactate contents and diamine oxidase activities were significantly increased in piglets of group Z (*p* < 0.01 or *p* < 0.05). Compare with Group Z, ileal villus height, lipase and α-amylase activities, occluding, claudin-1 and ZO-1 mRNA expressions, and plasma D-xylose levels were significantly elevated in piglets of group ZS1 and ZS2 (*p* < 0.01); while compare to Group Z, plasma D-lactate and diamine oxidase contents were significantly reduced in piglets of group ZS1 and ZS2 (*p* < 0.01 or *p* < 0.05). Compare with Group Z, value of villus height/crypt depth and trypsin activity were significantly promoted in piglets of group ZS2 (*p* < 0.01); whereas ileal crypt depth was significantly reduced in piglets of group ZS2 (*p* <0.01).Thus, SOC can mitigate ZEN-induced intestinal dysfunction in piglets.

## 1. Introduction

As a common mycotoxin in cereals and feed, zearalenone (ZEN) has a variety of toxic effects such as reproductive toxicity, enterotoxicity, immunotoxicity, genotoxicity and carcinogenicity, which may cause significant health effects on humans and livestock (1, 2). Among all livestock, pigs are the most sensitive to ZEN. ZEN can cause reproductive dysfunction in sows, decreased semen quality in boars, and reduced growth performance in piglets and fattening pigs, resulting in serious economic losses (3). More seriously, ZEN may remain in pork products after slaughter and processing, which may endanger human health (2, 4). Therefore, it is an urgent issue to mitigate ZEN-induced toxic effects in pig.

Selenized-chitosan is a new organic selenium compound obtained from chitosan and inorganic selenium by chemical synthesis method, which can simultaneously play the comprehensive role of chitosan and organic selenium (5). Studies found that selenized-chitosan could mitigate the damage of antioxidant capacity, regulate immune function, enhance disease resistance, improve the intestinal health, as well as promote production and growth performance of livestock (5, 6). Similarly, selenized-oligochitosan (SOC) is synthesized from oligochitosan with smaller molecular weight and inorganic selenium. SOC has better solubility and higher biological activity compared with ordinary selenized-chitosan due to its smaller relative molecular mass. Therefore, it has broad application prospects. Selenized-chitosan has been shown to antagonize the toxic effects of ZEN on animals. For example, Li et al. (2017) found that selenized-chitosan antagonized ZEN-induced changes in blood IL-18 and TNF-α contents in mice (7). Furthermore, Qin et al. (2022) also found that selenized-chitosan alleviated reduction of antioxidant capacity and immunosuppression induced by ZEN in mice (8). Prevenient study had shown that SOC was also effective in mitigating harmful effects induced by ZEN on piglets performance, blood biochemical indicators, antioxidant function and intestinal flora (9). Apart from intestinal flora, no studies have been reported on SOC alleviating ZEN-induced intestinal dysfunction in pigs; nor on chitosan or selenized-chitosan improving ZEN-induced intestinal dysfunction in pigs. Therefore, this trial investigated the effects of SOC in alleviating ZEN-induced damage to intestinal digestive, absorptive and barrier functions of piglets.

## 2. Materials and methods

### 2.1. Animals and handlings

Sixty (35 days old) ternary cross piglets were selected and randomly divided into 3 replicates of 4 groups (Group C, Z, ZS1 and ZS2). Group C was fed the basal diet (containing 0.06 μg/g selenium) as a control and Group Z was supplemented with 2 μg/g ZEN in the basal diet; Group ZS1 and ZS2 were supplemented with 0.3 or 0.5 μg/g SOC (calculated by selenium), respectively, in addition to 2 μg/g ZEN in the basal diet. The pre-trial period was 7 d and the trial period was 42 d. Food and water were taken *ad libitum* and routinely managed in the whole trial period.

### 2.2. Sampling and processing

On the morning of the last day of the trial, 2 piglets in each replicate were randomly selected and gavaged with 10% D-xylose solution (1 mg/kg BW), and blood samples were collected from the anterior vena cava one hour later in sodium heparin anticoagulation tubes, centrifuged and plasma collected for the determination of D-xylose, D-lactate contents and diamine oxidase activitie (10). Then the piglets were anaesthetised with intravenous sodium pentobarbital and the abdominal cavity was exposed and ileal samples were harvested. A portion of the samples were frozen in liquid nitrogen to measure mRNA expressions, and another portion of the samples were fixed in 4% paraformaldehyde to measure the intestinal mucosal structure. Ileum contents were also harvested in Eppendorf tubes for immediate determination of intestinal digestive enzyme activity.

### 2.3. Laboratory Analysis

#### 2.3.1. Determination of intestinal mucosal structure

The ileal samples fixed in 4% paraformaldehyde were routinely sectioned and stained, and the ileal mucosal structure (villus height and crypt depth) was determined in accordance with Wang et al. (2019) with CX31-DP72 microscopic imaging system (Olympus, Japan) (11). 15 images of a typical field of view (with the morphological integrity of the villus and crypt) were determined for each sample, and the average values were recorded, then the values of villus height/crypt depth were calculated.

#### 2.3.2. Determination of Ileal digestives enzyme activities

About 1 g of ileal contents was weighed, and 9 times the volume of pre-chilled saline was added to the contents and homogenized in an ice water bath. Then, 10% homogenates was obtained and centrifuged (3,200 r/min, 8 min) at 4°C in a freezing centrifuge to obtain supernatant, and the extracted supernatant was assayed for trypsin, lipase and α-amylase activities with appropriate kits (Nanjing Jiancheng Bioengineering Institute, China), respectively. Each measurement was performed strictly in duplicate to obtain average values.

#### 2.3.3. Determination of plasma D-lactate, diamine oxidase and D-xylose contents

D-lactate, D-xylose contents and diamine oxidase activities in piglet plasma were also measured with kits manufactured by Nanjing Jiancheng Bioengineering Institute. Each measurement was carried out in duplicate to obtain average values.

#### 2.3.4. Determination of Ileal tight junction protein mRNA expressions

Relative mRNA expressions of ileal tissue tight junction protein (TJP) ZO-1, occludin and claudin-1 were determined by the 2^−*ΔΔ*Ct^ method. The primers were synthesized by Beijing Biotechnology Co., Ltd. Upstream primer sequence of β-actin, ZO-1, occludin and claudin-1 were 5´-GATCTGGCACCACACCTTCTACAAC-3′, 5´-CCAGGGAGAGAAGTGCCAGTAGG-3′, 5´-CAGTGGTAACTTGGAGGCGTCTTC-3′ and 5´-AGAAGATGCGGATGGCTGTCATTG-3′, respectively. Downstream primer sequence of β-actin, ZO-1, occludin and claudin-1 were 5´-TCATCTTCTCACGGTTGGCTTTGG-3′, 5´-TTTGGTGGGTTTGGTGGGTTGAC-3′, 5´-CGTCGTGTAGTCTGTCTCGTAATGG-3′ and 5´-ACCATACCATGCTGTGGCAACTAAG-3′, respectively.

Total RNA extraction, reverse transcription of total RNA to cDNA and real-time quantitative PCR reactions for ileal samples were performed using TRIzol reagents (Invitrogen, UK), First Strand cDNA Synthesis Kit (Genecopoeia, USA) and SYBR Green qPCR mix 2.0 kit (Genecopoeia, USA), respectively. Real-time quantitative PCR was performed on a CFX96 Touch real-time PCR system (Bio-Rad, USA) under the following reaction conditions: initial denaturation at 95°C for 10 min; 44 cycles of denaturation at 95°C for 20 s, annealing at 60°C for 20 s and extension at 72°C for 15 s.

### 2.4. Statistical analysis

Results are expressed as means ± standard deviation. Data were analyzed using one-way ANOVA and the least significant difference method or Tamhane’s T2 test with SPSS 22.0 statistical analysis software (SPSS Inc., USA). Significance of differences in means between groups was determined at *p* < 0.05.

## 3. Results

### 3.1. Intestinal mucosal structure

**Figure 1:**
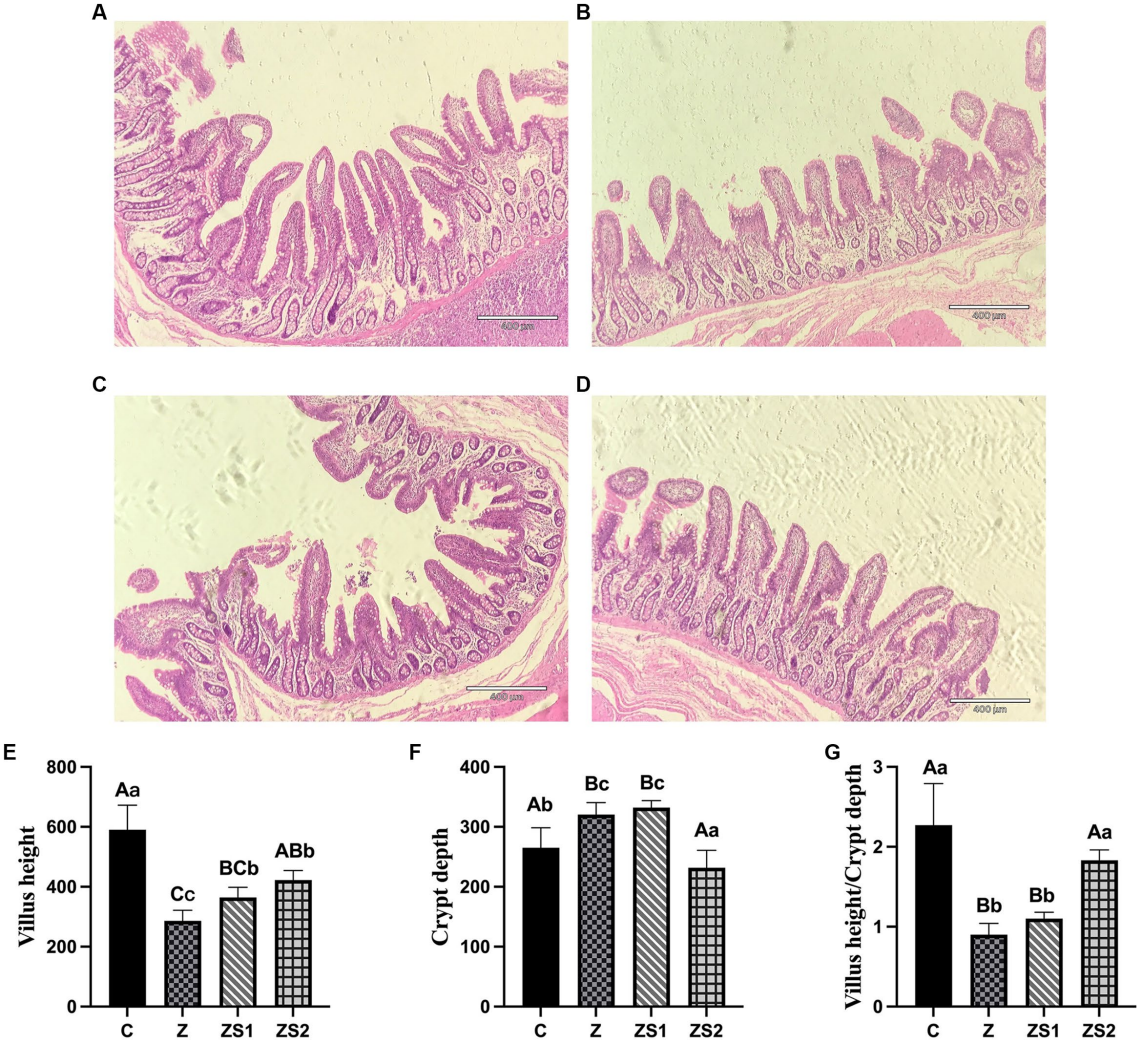
Effects of SOC on ileal morphology induced by ZEN in piglets: **(A)** ileal morphology in control; **(B)** ileal morphology in group Z; **(C)** ileal morphology in group ZS1; **(D)** ileal morphology in group ZS2; **(E)** Means of villus height; **(F)** Means of crypt depth; **(G)** Means of villus height/Crypt depth. Different capital letters (lowercase letters) in the column chart indicate a significant difference at the 0.01 (0.05) level.

The results were shown in Figure 1. Ileal villi and crypt were neatly arranged and no abnormal pathological changes were observed in piglets of group C (Figure 1A). The villi were not neatly arranged, not tightly packed, with large gaps between the villi and fractures, and the crypt was not neatly arranged in piglets of group Z; some of the ileal mucosal epithelial cells in piglets of group Z showed apoptosis, which was characterized by smaller cells, nuclear consolidation or lysis, enhanced cytoplasmic eosinophilia, and the presence of apoptotic vesicles (Figure 1B). Compare with group C, the height of the ileal villi and the value of villi height/crypt depth were significantly decreased (*p* < 0.01), while the crypt depth was significantly increased in piglets of group Z (*p* < 0.01). Compare with group Z, the villi height was promoted in piglets of group ZS1 (*p* < 0.05), while the differences in crypt depth and the value of villi height/crypt depth were not significant (*p* > 0.05). Compared with group Z, the ileal mucosa morphology in the ZS2 group was basically normalized, with improved villi breakage and neater crypt arrangement; decreased the number of apoptotic cells (Figure 1D), ileal villi height and value of villi height/crypt depth increased significantly (*p* < 0.01), while crypt depth decreased (*p* < 0.01). In addition, Compared with group ZS1, the value of villi height/crypt depth were also elevated (p < 0.01), while crypt depth was also reduced in piglets of group ZS2 (*p* < 0.01). However, there was no significant difference in ileal villi height of piglets between group ZS2 and ZS1 (*p* > 0.05).

### 3.2. Ileal digestive enzymes activities

**Figure 2:**
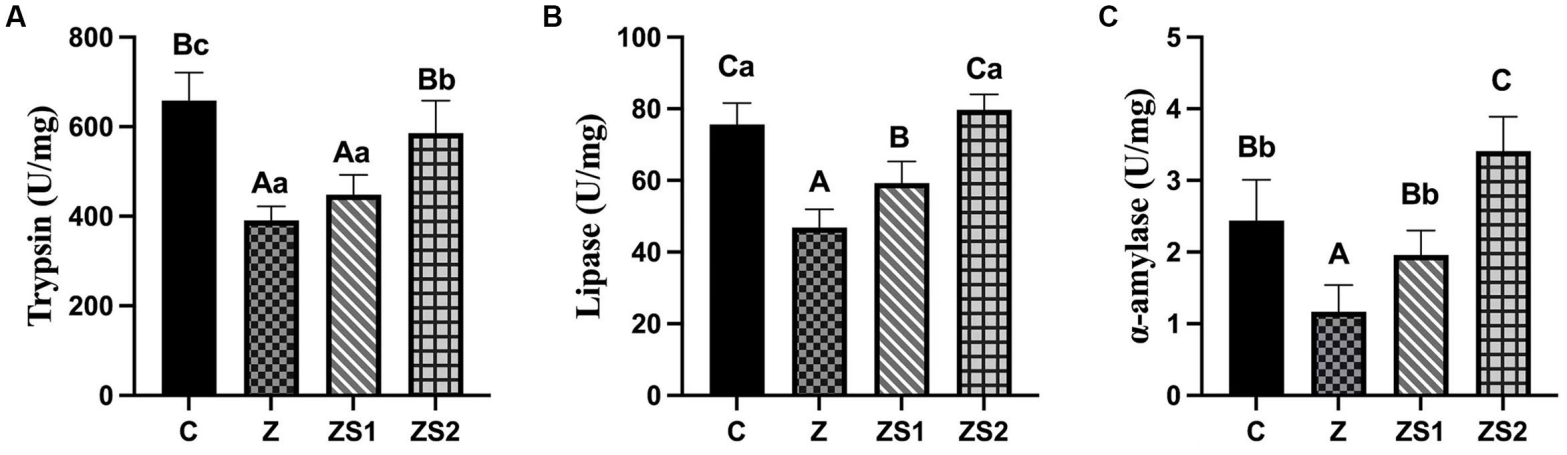
Effects of SOC on ileal digestive enzyme activities induced by ZEN in piglets: **(A)** Trypsin; **(B)** Lipase; **(C)** α-amylase. Different capital letters (lowercase letters) in the column chart indicate a significant difference at the 0.01 (0.05) level.

The results were shown in Figures 2A-C. Compare with group C, ileal trypsin, lipase and α-amylase activities were all decreased in piglets of group Z (*p* < 0.01). Compare with group Z, ileal lipase and α-amylase activities were all increased in piglets of group ZS1 and ZS2 (*p* < 0.01), and ileal trypsin activity was also increased in piglets of group ZS2 (*p* < 0.01). Compare with group ZS1, ileal trypsin, lipase and α-amylase activities were also improved in piglets of group ZS2 (*p* < 0.01).

### 3.3. Plasma D-lactate contents, D-xylose contents and diamine oxidase activities

**Figure 3:**
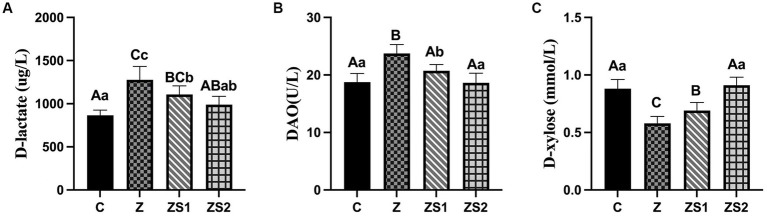
Effects of SOC on plasma levels of dierent parameters induced by ZEN in piglets: **(A)** D-lactate; **(B)** DAO; **(C)** D-xylose. Different capital letters (lowercase letters) in the column chart indicate a significant difference at the 0.01 (0.05) level.

The results were shown in Figures 3A-C. Plasma D-lactate contents and diamine oxidase activities of piglets were increased (*p* < 0.01) and the D-xylose contents of piglets were decreased (*p* < 0.01) in the Z group compare to the control group. However, plasma D-lactate contents and diamine oxidase activities of piglets were decreased (*p* < 0.05 or *p* < 0.01) and the D-xylose contents of piglets were increased (*p* < 0.01) in the ZS1 and ZS2 group compare to the Z group. Additionally, plasma diamine oxidase activities of piglets were also decreased (*p* < 0.05) and the D-xylose contents of piglets were also increased (*p* < 0.01) in the ZS2 group compare to the ZS1 group.

### 3.4. Ileal TJP mRNA expressions

**Figure 4:**
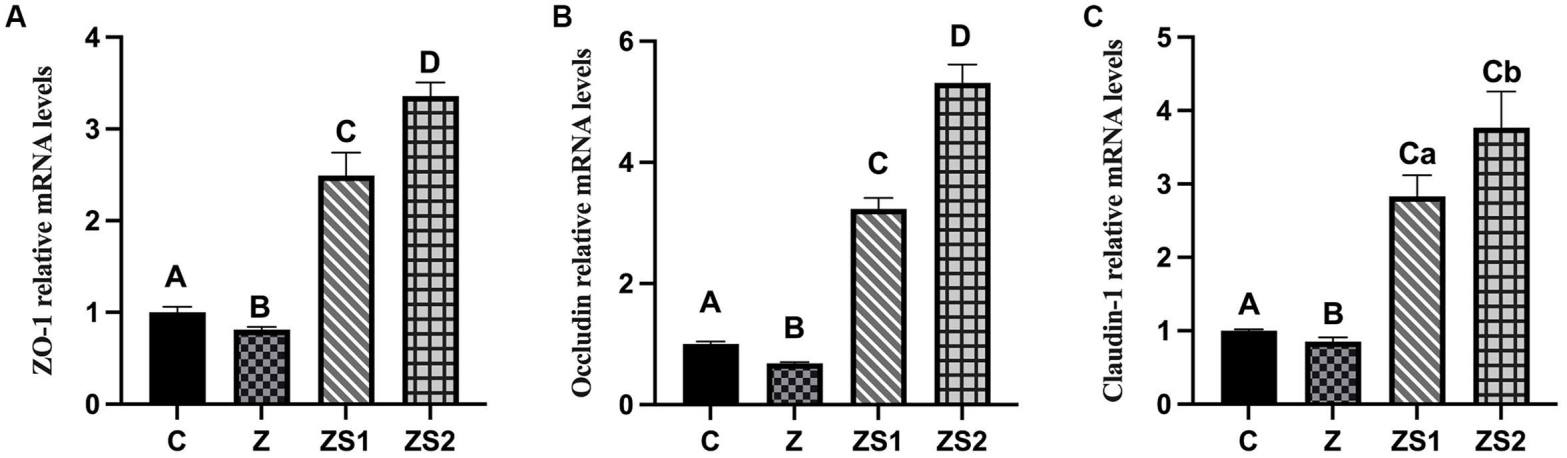
Effects of SOC on the ileal mRNA Expressions of TJPs induced by ZEN in piglets: **(A)** ZO-1; **(B)** Occludin; **(C)** Claudin-1. Different capital letters (lowercase letters) in the column chart indicate a significant difference at the 0.01 (0.05) level.

The results were shown in Figures 4A-C. The mRNA expressions of ZO-1, occludin and claudin-1 in ileum of piglets were decreased in the Z group compare to the control group (*p* < 0.01). However, The mRNA expressions of ileum ZO-1, occludin and claudin-1 of piglets in the ZS1 and ZS2 groups were greater than those in the Z group (*p* < 0.01). Additionally, the mRNA expressions of ileum ZO-1, occludin and caudin-1 of piglets were also increased in the ZS2 group compare to in the ZS1 group (*p* < 0.01 or *p* < 0.05).

## 4. Discussion

The intestine is in direct contact with ZEN in feed and is involved in ZEN absorption and metabolism (2), and thus ZEN could induce intestinal damage, which resulting in a reduction of absorption and barrier function in animals intestine (12, 13). The ileum is the last section of the small intestine, which is connected to the jejunum in front and the cecum in the back; the ileum can secrete digestive enzymes and active substances to play the role of digestion and absorption of nutrients. Although compared with jejunum and duodenum, ileum has a slightly lower nutrient absorption capacity, it is still one of the important absorption parts of the intestine; therefore, the ileum of piglets was chosen as the research object in this study. It is generally accepted that intestinal villus height, villus height/crypt depth value and crypt depth are usually used as important indicators to evaluate intestinal digestive and absorptive function (14). The results of this trial found that feeding a diet containing 2.0 μg/g ZEN for 42 days leaded to decrease in intestinal villus height and value of villus height /crypt depth and increase in crypt depth in the ileum of piglets; which indicated a change in the intestinal crypt-villus axis function (15). The decrease in intestinal villus height and value of villus height /crypt depth is usually considered to be related to insufficient cell proliferation in the intestinal crypt or excessive cell shedding in the villi (16, 17). For the present results, it may be mainly due to ZEN-induced apoptosis and consequently excessive cell shedding in the villi (18). The results of this trial also found that SOC improved the damage to the ileal villi of piglets by ZEN, as evidenced by restoration of villi alignment, decrease in crypt depth and increase in villi height, which may be the result of the simultaneous action of oligochitosan and organic selenium in SOC. Studies have shown that both organic selenium and chitosan have positive effects on mucosal structure of piglet intestine. Chitosan was able to increase the villi height and thevalue of villi height/crypt depth in the jejunum and ileum of piglets and decrease the crypt depth in the duodenum, jejunum and ileum (19). Organic selenium can also increase the villi height, reduce crypt depth and regulate stress-induced reduction in nutrient absorption capacity of piglets (20). Similar to the present tiral, Fang et al. (2018) found that organic selenium was able to reduce the damage of aflatoxin B1 to the intestine of broiler chickens, resulting in an improvement of intestinal villi height and villi height/crypt depth value, and a protective effect against intestinal damage (21).

Digestive enzymes are important active substances in animal’s intestine, which not only reflect the degree of absorption and /or digestion of nutrients, but also affect the growth, development and production performance in animals (22). Digestive enzymes in small intestine are mainly secreted by the stomach, pancreas and intestinal glands, of which amylase, protease and lipase activities are usually used as important indicators to assess the intestinal digestive function (23, 24). In this trial, SOC improved the ZEN-induced reduction in trypsin, lipase and α-amylase activities in the ileal contents of piglets, suggesting that SOC antagonized harmful effects to intestinal digestive function in piglets induced by ZEN; which could partially explain the mitigating effect of SOC on ZEN-induced reduction in piglet performance in previous study (9). And this may be largely the result of the role of chitosan in the SOC, which has been shown to promote the digestive enzymes activities in intestine or its contents in piglets and geese (25, 26); whereas no studies have been reported on selenium improving the intestinal digestive enzymes activities in animals.

Blood diamine oxidase, D-lactate and D-xylose levels are indicators to evaluate intestinal barrier function and absorption function (10, 27). Diamine oxidase is an intracellular enzyme found mainly in the upper villi of the intestinal mucosa. When the intestinal mucosa is damaged, its permeability increases, leading to the passage of diamine oxidase from the villi into the bloodstream, which in turn results in an inprovement in diamine oxidase activity in the blood circulation, thus indirectly reflecting the intestinal integrity and the degree of damage to the intestinal barrier in animals (28, 29).

D-lactate is one of metabolic products produced by bacteria in the intestine of animals; when the intestine is damaged, the mucosal villi epithelium is shed and the mechanical barrier of the mucosa are damaged, resulting in an increase in mucosal permeability. At this time, a large amount of D-lactate enters the bloodstream through the damaged mucosa, and since mammals lack the enzyme system to metabolize D-lactate, blood D-lactate content reflects the degree of damage to intestinal structure and changes of intestinal permeability (29, 30). In this experiment, both 0.3 and 0.5 μg/g of SOC increased the reduction of plasma diamine oxidase and D-lactate levels in ZEN-induced piglets, indicating that SOC improved intestinal permeability and protected against ZEN-induced impairment of intestinal barrier function in piglets. Similarly, low molecular weight selenium-aminopolysaccharide, which consisting of a low molecular weight aminopolysaccharide (derived from chitosan) and organic selenium, attenuated oxidative stress on intestinal permeability and intestinal barrier function in piglets, and antagonized diamine oxidase activity and d-lactate concentrations in plasma (31). In addition, organic selenium could reduce oxidative stress-induced changes in blood diamine oxidase activity and D-lactate concentrations in pigs, reducing the damage of intestinal mucosal and protecting the integrity of intestinal barrier (32). Furthermore, studies have shown that oligochitosan was also able to increase blood D-lactate concentrations and diamine oxidase activity and villi height in jejunum and ileum in stressed rats, reducing intestinal permeability and protecting intestinal barrier function (33, 34).

D-xylose absorption test is one of the effective methods to evaluate the intestinal absorption function of animals, which can reflect the intestinal absorption capacity by detecting the degree of D-xylose absorption in the small intestine, i.e., a higher blood D-xylose concentration indicates better intestinal digestion and absorption (10, 35). In this trial, both 0.3 and 0.5 μg/g of SOC reduced the ZEN-induced increase in plasma D-xylose levels in piglets, suggesting that SOC alleviated the harmful effects to intestinal absorption function in piglets induced by ZEN, which could also partially explain the improvement of ZEN-induced reduction in piglet performance by SOC found in previous study (9).

Tight junctions are the primary mode of attachment between intestinal mucosal epithelial cells, which are mainly composed of junctional complex proteins (zonula occludens, cingulin, symplekin, etc.), transmembrane proteins (Occludin, tricellulin, Claudins, etc.) and cytoskeletal structures (36, 37). TJPs are important indicators of the function of the physical barrier and permeability of the intestine. Among them, zonula occludens 1 (ZO-1), occludin and claudins have important roles in regulating intestinal permeability and maintaining integrity of intestinal tight junctions (37, 38). In the present trial, 2.0 μg/g of ZEN significantly downregulated the mRNA expression levels of ileal ZO-1, occludin and claudin-1 in piglet, indicating that ZEN caused harmful effects to the ileal mechanical barrier and increased intestinal permeability in piglets. The mechanism by which ZEN reduces transcription and translation of TJPs in the piglet intestine and thus damages the physical barrier of the piglet intestine may be related to the following factors. 1) ZO-1 not only binds to the cytoskeleton (microtubules, microfilaments and filaments), ocludin and claudin-1, but also regulates the assembly of the cytoskeleton at cell junctions (39), whereas ZEN induces significant downregulation of mRNA expression in TJP, and also induces redistribution of the ZO-1, which altered the tight junction structure and improved intestinal permeability (40). 2) ZEN disrupts the TJP structure by activating RhoA/ROCK signaling pathway resulting in rearrangement of cellular microfilaments and alteration of the cytoskeleton, which in turn damages intestinal physical barrier and increases intestinal permeability (40). 3) ZEN impedes the repair of intestinal epithelial and mucosal damage in piglets by preventing the expression of TGF-β1 and Smad family proteins in TGF-β1/Smads signaling pathway (41).

In this experiment, both 0.3 and 0.5 μg/g of SOC increased the reduction of mRNA expressions of ZO-1, occludin and claudin-1 induced by ZEN in piglet, indicating that SOC improved intestinal permeability and reduced ZEN-induced harmful effects to the intestinal physical barrier in piglets. Similarly, low molecular weight selenium-aminopolysaccharide was able to significantly increase the expressions of ileal ZO-1 and occludin in weaned piglets, protecting ileal tight junctions and reducing ileal intestinal permeability (31). However, the mechanism of how SOC mitigated the effects of ZEN on TJPs, and thus improved intestinal permeability and attenuated ZEN-induced physical barrier damage in piglets (e.g., whether it activated the TGF-β1/Smads signaling pathway and/or prevented the RhoA/ROCK signaling pathway) still needs further investigation.

## 5. Conclusion

SOC antagonized ZEN-induced changes in ileal mucosal structure, digestive enzyme activity, permeability, and tight junction protein mRNA expression levels in piglets, indicating that it protected against ZEN-induced impairment of intestinal digestive, absorptive and barrier functions in piglets.

## Data availability statement

The original contributions presented in the study are included in the article/supplementary materials, further inquiries can be directed to the corresponding author.

## Ethics statement

The animal study was reviewed and approved by Experimental Animal Ethics Committee of Tianjin Agricultural University.

## Author contributions

SQ: investigation, data analysis, validation, and writing—original draft and editing. YP: investigation and data analysis. FS: investigation and validation. JZ: supervision and resources. LL: supervision and project administration. FC: writing—review and editing, supervision, and funding acquisition. All authors contributed to the article and approved the submitted version.

## Funding

This research was supported by Key Project of Tianjin Natural Science Foundation (20JCZDJC00170), Shandong Natural Science Foundation (ZR2021MC150), Shandong Science and Technology Small and Medium Enterprises Innovation Ability Improvement Project (2021tsgc1303), and Shandong Modern Agricultural Technology and Industry System (SDAIT-11-07).

## Conflict of interest

The authors declare that the research was conducted in the absence of any commercial or financial relationships that could be construed as a potential conflict of interest.

## Publisher’s note

All claims expressed in this article are solely those of the authors and do not necessarily represent those of their affiliated organizations, or those of the publisher, the editors and the reviewers. Any product that may be evaluated in this article, or claim that may be made by its manufacturer, is not guaranteed or endorsed by the publisher.

## Supplementary material

The Supplementary material for this article can be found online at: https://www.frontiersin.org/articles/10.3389/fvets.2023.1184969/full#supplementary-material
