# Supplementary material for: Positive effects of selenized-oligochitosan on zearalenone-induced intestinal dysfunction in piglets
Source: Front Vet Sci. 2023 May 16;10:1184969. doi: 10.3389/fvets.2023.1184969 (PMC10228365; doi:10.3389/fvets.2023.1184969)
Supplement: Supplementary file 1 [file Table_1.DOCX]

Supplementary Material

Positive effects of selenized-oligochitosan on zearalenone-induced intestinal dysfunction in piglets

Shunyi Qin, Yukai Peng, Fuze She, Jianbin Zhang, Liuan Li and Fu Chen^*^

*** Correspondence:** Fu Chen: cf507@sohu.com

# Supplementary Tables

Table 1. Details of the primers

| Genes | Product length | Primer sequence | Genebank Access number |
| --- | --- | --- | --- |
| β-actin | 107 bp | F:5´- GATCTGGCACCACACCTTCTACAAC-3´  R:5´- TCATCTTCTCACGGTTGGCTTTGG-3´ | AY550069.1 |
| ZO-1 | 92 bp | F:5´- CCAGGGAGAGAAGTGCCAGTAGG-3´  R:5´- TTTGGTGGGTTTGGTGGGTTGAC-3´ | XM_021098827.1 |
| Occludin | 103bp | F:5´- CAGTGGTAACTTGGAGGCGTCTTC-3´  R:5´- CGTCGTGTAGTCTGTCTCGTAATGG-3´ | NM_001163647.2 |
| Claudin-1 | 83bp | F:5´- AGAAGATGCGGATGGCTGTCATTG-3´  R:5´- ACCATACCATGCTGTGGCAACTAAG-3´ | NM_001244539.1 |

Table 2. Data sheet of figure 1.

| Groups | Villus height | Crypt depth | Villus height/Crypt depth |
| --- | --- | --- | --- |
| C | 590.03±82.25 Aa | 265.20±33.34Ab | 2.27±0.52Aa |
| Z | 286.47±34.87Cc | 320.57±19.88Bc | 0.90±0.14Bb |
| ZS1 | 364.03±34.40BCb | 332.07±11.74Bc | 1.10±0.08Bb |
| ZS2 | 422.25±32.31ABb | 231.88±28.79Aa | 1.83±0.13Aa |

Different capital letters (lowercase letters) in the column chart indicate a significant difference at the 0.01 (0.05) level. The same as follows.

Table 3. Data sheet of figure 2.

| Groups | Trypsin (U/mg) | Lipase (U/g) | α-amylase (U/mg) |
| --- | --- | --- | --- |
| C | 658.38±62.33Bc | 75.63±6.02Ca | 2.44±0.57Bb |
| Z | 390.96±31.46Aa | 46.85±5.10A | 1.17±0.37A |
| ZS1 | 448.64 ±43.51Aa | 59.29±6.01B | 1.96±0.34Bb |
| ZS2 | 585.34±73.15Bb | 79.68±4.38Ca | 3.41±0.48C |

Table 4. Data sheet of figure 3.

| Groups | D-lactate (ug/L) | DAO(U/L) | D-xylose (mmol/L) |
| --- | --- | --- | --- |
| C | 866.58±59.07Aa | 18.76±1.50Aa | 0.88±0.08Aa |
| Z | 1277.95±153.73Cc | 23.72±1.54B | 0.58±0.06C |
| ZS1 | 1107.54±99.53BCb | 20.73±1.09Ab | 0.69±0.07B |
| ZS2 | 988.71±96.90ABab | 18.64±1.67Aa | 0.91±0.07Aa |

Table 5. Data sheet of figure 4.

| Groups | ZO-1 | Occludin | Claudin-1 |
| --- | --- | --- | --- |
| C | 1.00±0.06A | 1.00±0.04A | 1.00±0.02A |
| Z | 0.81±0.03B | 0.68±0.02B | 0.85±0.06B |
| ZS1 | 2.49±0.25C | 3.23±0.18C | 2.83±0.29Ca |
| ZS2 | 3.36±0.15D | 5.31±0.31D | 3.77±0.49Cb |
